# Beat-to-Beat Continuous Blood Pressure Estimation Using Bidirectional Long Short-Term Memory Network

**DOI:** 10.3390/s21010096

**Published:** 2020-12-25

**Authors:** Dongseok Lee, Hyunbin Kwon, Dongyeon Son, Heesang Eom, Cheolsoo Park, Yonggyu Lim, Chulhun Seo, Kwangsuk Park

**Affiliations:** 1 Interdisciplinary Program in Bioengineering, Seoul National University, Seoul 03080, Korea; azuremoon@bmsil.snu.ac.kr (D.L.); chasekwon@bmsil.snu.ac.kr (H.K.); dongyeonson@bmsil.snu.ac.kr (D.S.); 2 Medical Research Center, Institute of Medical and Biological Engineering, Seoul National University, Seoul 03080, Korea; 3 Department of Computer Engineering, Kwangwoon University, Seoul 01897, Korea; surmounting@kw.ac.kr (H.E.); parkcheolsoo@kw.ac.kr (C.P.); 4 Department of Oriental Biomedical Engineering, Sangji University, Wonju 26339, Korea; yglim@sangji.ac.kr; 5 School of Electronic Engineering, Soongsil University, Seoul 06978, Korea; chulhun@ssu.ac.kr; 6 Department of Biomedical Engineering, College of Medicine, Seoul National University, Seoul 03080, Korea

**Keywords:** cuffless blood pressure, ballistocardiogram, long short-term memory, general blood pressure estimation

## Abstract

Continuous blood pressure (BP) monitoring is important for patients with hypertension. However, BP measurement with a cuff may be cumbersome for the patient. To overcome this limitation, various studies have suggested cuffless BP estimation models using deep learning algorithms. A generalized model should be considered to decrease the training time, and the model reproducibility should be taken into account in multi-day scenarios. In this study, a BP estimation model with a bidirectional long short-term memory network is proposed. The features are extracted from the electrocardiogram, photoplethysmogram, and ballistocardiogram. The leave-one-subject-out (LOSO) method is incorporated to generalize the model and fine-tuning is applied. The model was evaluated using one-day and multi-day tests. The proposed model achieved a mean absolute error (MAE) of 2.56 and 2.05 mmHg for the systolic and diastolic BP (SBP and DBP), respectively, in the one-day test. Moreover, the results demonstrated that the LOSO method with fine-tuning was more compatible in the multi-day test. The MAE values of the model were 5.82 and 5.24 mmHg for the SBP and DBP, respectively.

## 1. Introduction

Blood pressure (BP) is one of the most important physiological signals that indicates fundamental health information of the patient. When the heart beats, the BP varies between systolic BP (SBP) and diastolic BP (DBP). An estimated 1.13 billion people worldwide have high blood pressure (hypertension), which is known as a high risk factor for various diseases such as heart attack, blindness, and brain stroke.

The gold standard for measuring BP is arterial BP (ABP), by means of which the BP is measured directly from an intravascular cannula needle. ABP is accurate and continuous; however, this method is usually performed in intensive care units because it is invasive and requires a clinical setting. Moreover, it is difficult to measure the ABP in daily life.

Several methods have been developed to monitor the BP regularly, because frequent BP monitoring is important for the diagnosis of hypertension and the prediction of heart diseases. Although the oscillometric method is an easy-to-use BP monitoring technique, it does not offer continuous measurement [1]. To overcome this limitation, a continuous BP monitoring device was developed. However, both of these methods require an inflatable upper-arm cuff, which may cause the patient discomfort [2].

To address the above problem, many researchers have investigated cuffless and continuous BP monitoring methods based on the pulse wave velocity (PWV), which can be measured with physiological signals. The PWV can be expressed by the Moens–Korteweg (M–K) Equation (1) and Hughes Equation (2) [3]:

(1) $$PWV=\sqrt{\frac{Eh}{\rho d}}$$

(2) $$E=E_{0}e^{\gamma P},$$

where *E* is the elastic modulus at the BP *P*, ρ denotes the density of the blood, and *h* and *d* are the thickness and radius of the blood vessel, respectively. Furthermore, $E_{0}$ is the elastic modulus at zero BP and γ is the coefficient of the blood vessel. When the blood pressure *P* increases, the elastic modulus increases, and thus, the PWV also increases.

(3) $$\mathrm{PWV}=\frac{L}{PTT},$$

PWV is inversely related to the pulse transit time (PTT), as follows: where *L* denotes the length from the heart to a certain peripheral site of the body such as the finger. The PTT is the time taken by the pulse to propagate from two locations. The PTT can be calculated between the R-peak of the electrocardiogram (ECG) and the characteristic point of the photoplethysmogram (PPG) that is measured at the finger. The ECG and PPG are measured using a noninvasive method and can easily be used in long-term monitoring or daily life without a cuff. The PTT is known to be negatively correlated with the BP [4], and various models have been developed to estimate the BP with the PTT [5,6,7]. However, Payne et al. reported that the PTT method is not a reliable marker for BP estimation [8].

Several studies have suggested the ballistocardiogram (BCG) as a substitute for the PPG in calculating the PTT. The BCG is a measurement of the forces exerted by the blood flow ejected from the heart on the body. The BCG can be acquired by force sensors such as accelerometers, load cells, and film sensors including polyvinylidene fluoride (PVDF) sensors. Shin et al. proposed a BP measurement system using ECG and BCG on a weighing scale [9]. The BCG was measured on the weighing scale, and the RJ interval (RJI) between the R-peak of the ECG and J-peak of the BCG was measured. The results demonstrated that the RJI had a negative correlation with the BP and the BP was estimated using the linear regression method. Lee et al. suggested a BP monitoring chair using two-channel BCGs [10]. Two BCGs were measured at the back of the chair and the cushion on the seat, and the BP was estimated according to the phase difference of the two BCGs.

In recent studies, deep learning algorithms, including convolutional neural networks (CNNs), have been applied in biomedical fields such as image classification and signal pattern extraction [11,12,13]. Certain researchers have applied deep learning algorithms to continuous BP estimation using physiological signals such as ECG and PPG. Wu et al. proposed a deep neural network (DNN) based on combined information using ECG and PPG [14]. Moreover, Li et al. suggested a real-time BP estimation model with a long short-term memory (LSTM) network using the features of ECG and PPG [15]. In our previous study, we investigated an end-to-end BP estimation algorithm using a CNN with an attention mechanism [16]. The results revealed that the BCG signal with ECG and PPG exhibited superior performance in BP estimation. However, the algorithm was based on whole signals, including noise signals, and only a person-specific model was described.

When developing a deep learning model, a generalized model should be considered for application to real situations because the training time of the network is long. Furthermore, the reproducibility of the model in a multi-day situation is an important factor for continuous BP estimation. In this study, we developed a feature-based deep learning algorithm using a bidirectional LSTM network to improve the performance. Furthermore, a general BP estimation model using multiple measurement data was considered for robust reproducibility.

## 2. Materials and Methods

**Figure 1:**
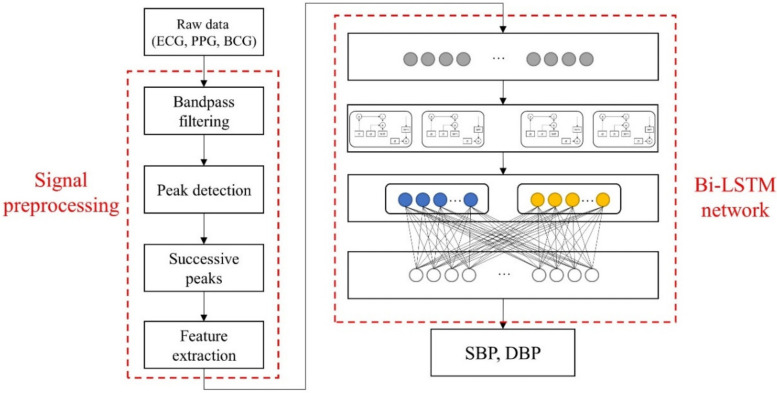
Overview of proposed approach.

An overview of our proposed approach is illustrated in Figure 1. The approach comprises two parts, signal preprocessing and BP estimation using bi-LSTM network. The methods are detailed in the following subsections.

### 2.1. Data Acquisition

A total of 18 subjects (male: 8, female: 10) with no medical records reported were recruited for the experiment. Written informed consent was obtained from the subjects, and the study was approved by the Institutional Review Board of Seoul National University Hospital (IRB No. 1801-016-912).

Several devices were attached to the subject to measure physiological signals. Three Ag/AgCl electrodes were attached to the subject according to Einthoven’s triangle, and the ECG was acquired on lead II with the BIOPAC ECG100C module. The PPG was measured from the index finger of the subject using a commercial module (PSL-iPPG2C), whereas the BCG signal was measured from the PVDF sensor (Measurement Specialties, Hampton, VA, USA) installed on the chair seat. The reference SBP and DBP were measured with a continuous BP monitoring device (Finometer^®^ PRO, Finapres Medical Systems, Enschede, The Netherlands). Once the devices were attached, the subject was asked to sit on the chair with the PVDF sensor and the signal was recorded for 30 min. All of the data were synchronized and digitized at 1000 Hz using a data acquisition device (BIOPAC MP150). Furthermore, 15 subjects visited again in one to two weeks and the measurement procedure was repeated with the same experimental setup.

### 2.2. Signal Preprocessing and Feature Extraction

A second-order Butterworth filter was applied to the signal to remove baseline wandering, motion artifacts and power-line noise (ECG: 0.5 to 35 Hz; BCG: 4 to 15 Hz; PPG: 0.5 to 8 Hz). The characteristic points from the ECG, BCG, and PPG were used to extract the features. First, the R-peak of the ECG was detected using the Pan–Tompkins algorithm. The J-peak of the BCG was detected by identifying the highest peak between 110 and 250 ms after each R-peak. The PPG was differentiated to obscure motion artifacts and identify the peak of the first derivative of PPG (dPPG). After the peaks of the signal were detected, false-positive peaks were manually excluded and the features of each cardiac cycle were extracted.

**Figure 2:**
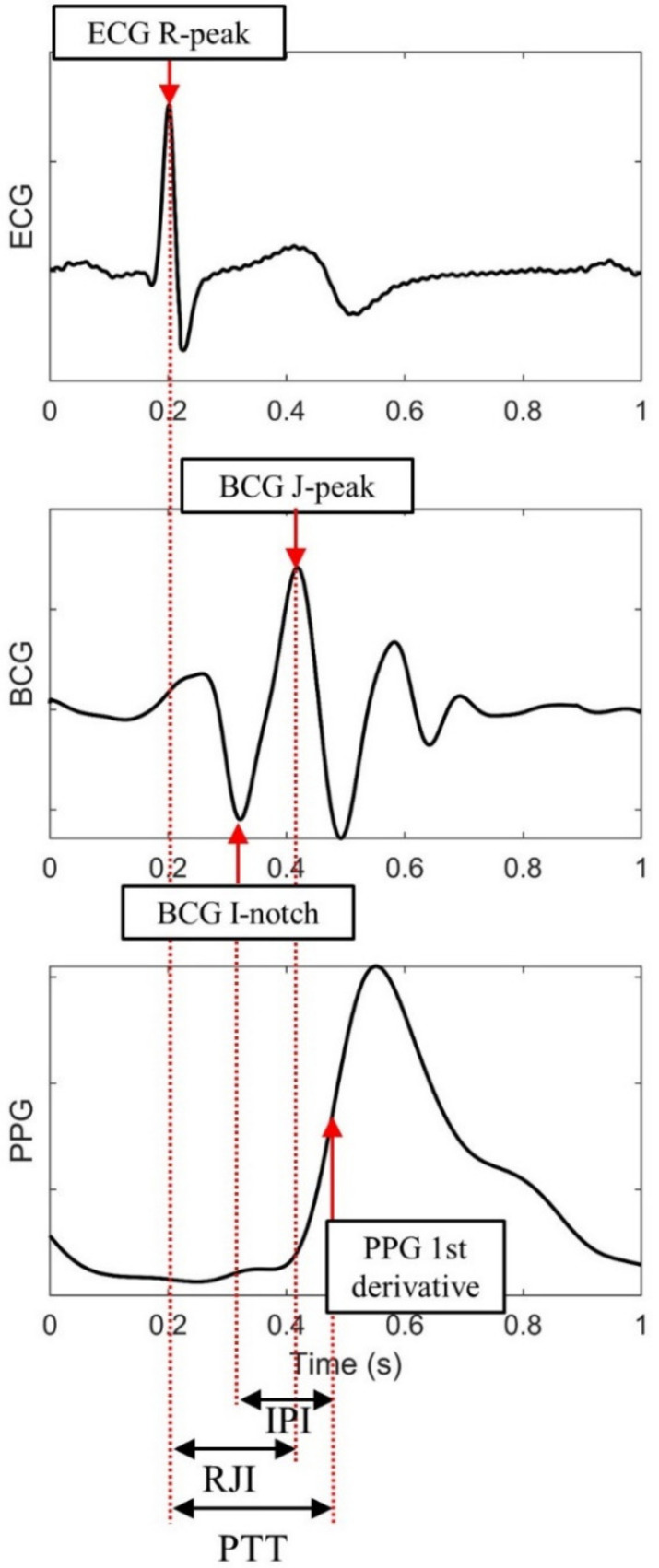
Feature extraction from characteristic points of three signals: electrocardiogram (ECG), ballistocardiogram (BCG), and photoplethysmogram (PPG).

**Figure 3:**
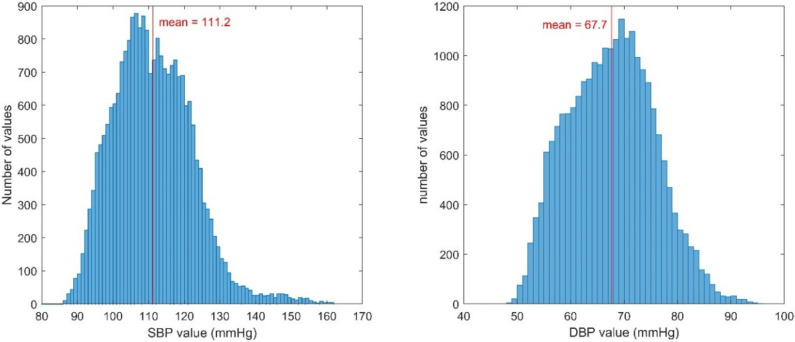
Blood pressure (BP) distributions of BPs in our data. (**left**) Systolic BP, (**right**) Diastolic BP.

The features are listed in Table 1 and the feature extraction method is depicted in Figure 2. The interval values (RRI, PTT, RJI, and IPI) between the characteristic points of the three signals and each amplitude of the peak of the signal (ECGamp, BCGamp, and PPGamp) were extracted as input features. Thereafter, the features were standardized with the mean and standard deviation values to be used as input for the neural network model. The features from 10 successive cardiac cycles were regarded as one sequence. The number of the cardiac cycles was determined empirically. If a sequence included undetected peaks, it was excluded. The SBP and DBP values immediately after the last peak in the sequence were used as reference and labeled for the output of the model. The SBP and DBP distributions are illustrated in Figure 3. The average values were 111.2 and 67.7 mmHg for the SBP and DBP, respectively.

### 2.3. Deep Learning

#### 2.3.1. LSTM Network

Recurrent neural networks (RNNs) have been demonstrated to offer high performance in time-series data. However, conventional RNNs suffer from the vanishing gradient problem, especially when handling long time-series data. An LSTM network was proposed to overcome the limitations of conventional RNNs [17]. The LSTM network replaces the RNN cells with LSTM cells. The LSTM cell has three gates: a forget gate, an input gate, and an output gate. The forget gate controls how much information will be forgotten using the hidden state and input vector. The input gate determines which value will be updated and subsequently updates the state of the cell. The output gate controls how much information is outputted. These gates can aid the network in learning long time-series data or eliminating meaningless data, and thus, learn patterns with a long duration. Bidirectional LSTM (Bi-LSTM) is an extension of LSTM in which the input sequence is read forward and backward, and both outputs are concatenated. Bi-LSTM is more powerful than LSTM because it can learn the pattern in both directions.

#### 2.3.2. Proposed Model Architecture

**Figure 4:**
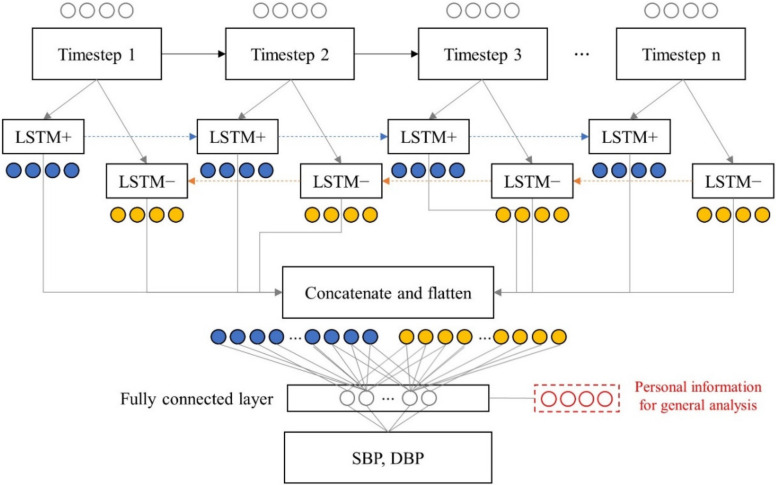
Diagram of proposed model architecture.

The proposed network architecture is summarized in Figure 4. The model consisted of a Bi-LSTM network and two fully connected layers. As the input comprised 10 cardiac cycles with 7 features, the shape of the input layer was 10 × 7. The number of hidden nodes of the Bi-LSTM network was empirically set to 128, and 256 features were generated at each timestep. The tanh function was used as the activation function of the LSTM layer. The outputs of the forward and backward LSTM cell were concatenated (10 × 256 neurons) and transformed into a one-dimensional layer (1 × 2560 neurons) with flatten layer for connecting to the fully connected layer after the LSTM layer. In the case of the general model, personal information including gender, age, height, weight, and body mass index (BMI) was included in the first fully connected layer. The second fully connected layer was used for the BP regression. ReLU and linear activation function were utilized at the first and second fully connected layers, respectively. The number of hidden nodes in the first fully connected layer was set to 64.

The deep learning model was implemented in the Keras framework with a TensorFlow backend. The data were shuffled and randomly selected to train the deep learning model. In total, 60% of the data was used for training, 20% was used for validation, and 20% was used for testing. The Adam optimizer was used to optimize the model with a learning rate of 10^−3^. The initial value was randomly determined and the mean squared error (MSE) was selected as the loss function. To address overfitting, a regularization method was adopted with a dropout mask on 10% of the connections in the LSTM layer. The model was trained with the early stopping method; patience was set to 10 for maximum of 100 training epochs. The batch size was set to 64.

Following the training process, the test set was used to estimate the SBP and DBP. The model was trained three times with different random initial values and the regression result was averaged. The correlation coefficient (CC), mean absolute error (MAE), and root mean squared error (RMSE) between the estimated and reference BPs, were calculated to evaluate the performance of the algorithm.

## 3. Results

### 3.1. Feature Analysis

The performance of the model with different inputs was evaluated. The features that were used as inputs are described in Table 2. As indicated in Table 3, the model with all three signals exhibited better performance than the other models. The MAEs were 2.62 and 2.03 mmHg, whereas the CC values were 0.77 and 0.76, for the SBP and DBP estimations, respectively. The difference between the models was statistically significant (*p* < 0.01).

### 3.2. General Model Analysis

Leave-one-subject-out (LOSO) analysis was performed to create a general model. The data of one subject were removed from the training set and the data of the other subjects were used as input to train the model. Moreover, a fine-tuning approach was applied after each training run. The weight in the Bi-LSTM layer was not trained and the fully connected layer was trained with 20% of the data of the excluded subject.

The results are summarized in Table 4. The MAE values of the LOSO model were 10.01 and 5.64 mmHg for the SBP and DBP, respectively. The LOSO model exhibited a higher error than the personal model (*p* < 0.01). The tuned LOSO model yielded MAE values of 2.56 and 2.06 mmHg for the SBP and DBP, respectively. It exhibited a slightly lower error than the personal model in the SBP, but a higher error in the DBP. The difference was not statistically significant.

**Figure 5:**
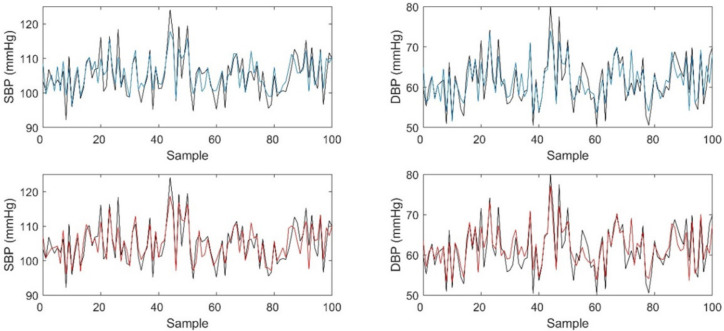
Comparison of reference and estimated BPs (black line: reference BP; blue line: personal model; red line: tuned leave-one-subject-out (LOSO) model).

A comparison of the personal and tuned LOSO models is presented in Figure 5. Although the difference was not statistically significant, the tuned LOSO model exhibited better performance than the personal model when the reference BP value was extremely high or low. Moreover, following the model creation, the tuned LOSO model requires fewer parameters to be trained and the model can be trained with a smaller amount of data, which requires less time.

**Figure 6:**
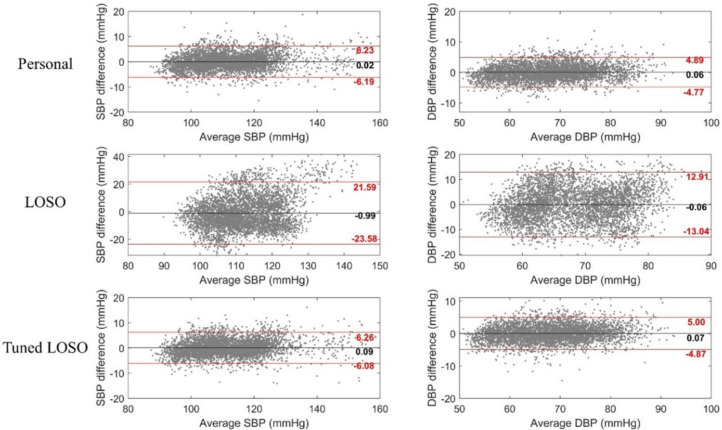
Bland–Altman plots of models. Black line: mean value; red line: 95% confidence interval.

Bland–Altman plots of the models are depicted in Figure 6. The bias was not significant in all three models, and the limits of agreement at a 95% confidence interval of the tuned LOSO model were [−6.08, 6.26] and [−4.87, 5.00] for the SBP and DBP, respectively.

### 3.3. Reproducibility Analysis

The model reproducibility had to be investigated to evaluate the model generalization. A multi-day test was performed using second visit data in addition to a one-day test. The model was trained with the data of one visit, and the data of the other visit was used as a test set. The results are presented in Table 5. The error was higher than the test results with only the first visit in the personal and tuned LOSO models. The MAE values of the tuned LOSO model were 5.82 and 5.24 mmHg for the SBP and DBP estimations, respectively. Although the comparison result between the personal and tuned LOSO models presented in Section 3.2 was not significant, the reproducibility of the tuned LOSO model was better than that of the personal model (*p* < 0.05). This is because the personal model was overfitted with the one-day condition of the subject.

**Figure 7:**
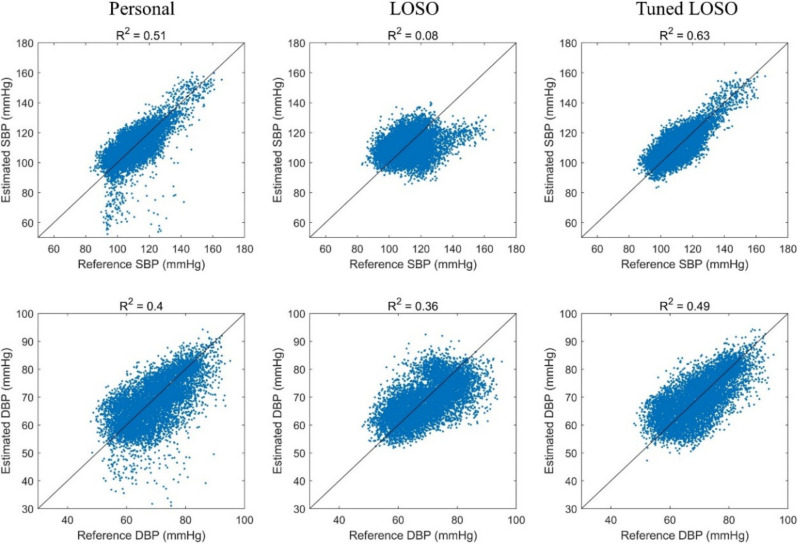
Scatter plots of reproducibility analysis.

Scatter plots for the model results are presented in Figure 7, with the coefficient of determination (R^2^) indicated. The personal model tended to underestimate the BP, and the R^2^ values were 0.51 and 0.4 for the SBP and DBP, respectively. The R^2^ values of the tuned LOSO models were 0.63 and 0.49 for the SBP and DBP, respectively. This means that the model learned more general patterns to estimate the BP than the personal model, with high reproducibility.

### 3.4. Evaluation Using International Standard

The proposed model of one-day and multi-day tests was evaluated using two international standards of BP estimation: the British Hypertension Society (BHS) standard [18] and the Association for the Advancement of Medical Instrumentation (AAMI) standard. The evaluation results are presented in Table 6. The BHS standard evaluates the BP estimation device based on the cumulative percentage of absolute errors under thresholds of 5, 10, and 15 mmHg. According to the BHS Standard, the proposed model was consistent, with grade A in the one-day test and grade B in the multi-day test for the SBP and DBP.

The evaluation results using the AAMI standard are described in Table 7. The AAMI standard requires mean error (ME) values lower than 5 mmHg and standard deviation (STD) values lower than 8 mmHg. According to the AAMI, the number of populations should be at least 85. Although this study did not satisfy the population criterion, both models satisfied the ME and STD values in the SBP and DBP estimation.

## 4. Discussion

To evaluate the performance of the proposed method, the algorithm was compared with three representative BP estimation methods proposed by Chen et al. [5], Poon et al. [6], and Ding et al. [7]. Further, our method was also compared against a conventional multiple linear regression (MLR) method with features that were used in the proposed model. Comparison results presented in Table 8 suggest that the model based on the pulse intensity ratio (PIR) exhibits the lowest error among the previous methods. The difference between the PIR model and MLR model without BCG features was not significant;however, the performance was markedly improved in the MLR model with the BCG features. The MAE of the MLR model with BCG features was 4.17 mmHg and 3.12 mmHg for SBP and DBP respectively. In addition, the MLR model, which utilized the features of previous 10 cardiac cycles exhibited better performance than the model with only one cardiac cycle. The proposed model showed the lowest error and provided a nonlinear expression between the features and the target BP.

Furthermore, the proposed model was compared with similar works using deep learning. The comparison results are summarized in Table 9. It was difficult to perform a fair comparison with other studies because the datasets used in the studies may differ significantly, and the validation methods also vary. Kachuee et al. [19], Slapničar et al. [20], and Hsu et al. [21] used an online database named “Medical Information Mart for Intensive Care unit (MIMIC)” [22]. This database contains a large number of clinical data, including those of ECG, breathing, PPG, and BP. However, the data may not be compatible for normal people because they were obtained from patients in intensive care units, and the patients could have been influenced by drugs that could affect the BP variation.

Kachuee et al. suggested a continuous BP estimation algorithm based on AdaBoost, but the error was relatively higher than that in other studies [19]. Slapničar et al. implemented a network architecture using a ResNet and spectro-temporal block, and performed LOSO analysis with the data [20]. Hsu et al. [21] and Wu et al. [14] proposed DNN models, in which the error was lower than that in other studies; however, they applied 10-fold cross-validation, which is different from our LOSO analysis. Su et al. proposed a long-term BP prediction model using a Bi-LSTM network [23]. The multi-day analysis was performed on the second and fourth days, and at six months, and the MAE values were 5.81 and 5.21 mmHg for the SBP and DBP, respectively. Although the error value was lower than that of our model, the validation was only performed with a personalized model. The BP estimation performance was enhanced compared to our previous work using a CNN and an attention mechanism.

**Figure 8:**
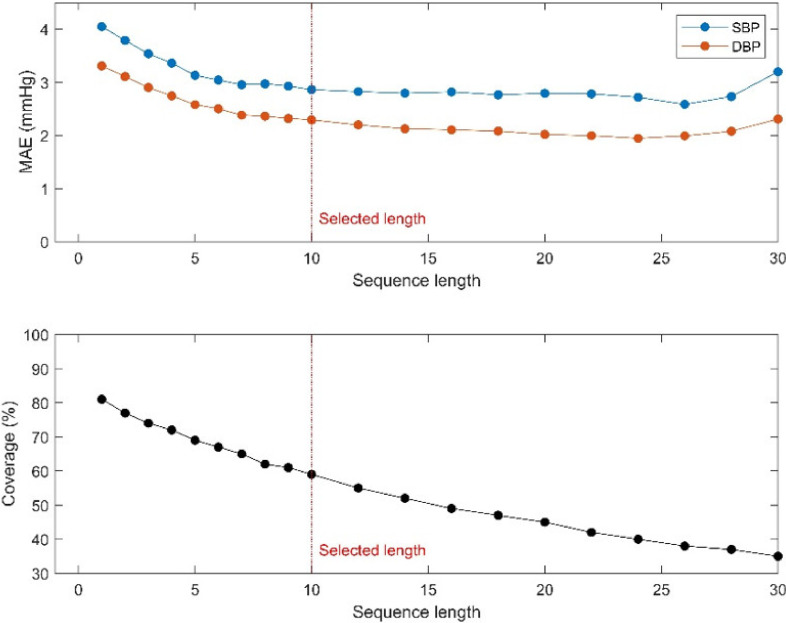
MAE values and coverage with different sequence lengths.

Figure 8 plots the performance of the model and coverage for different sequence lengths. The MAE value reduced until one sequence was generated with approximately 10 cardiac cycles and saturated until the sequence length was 25. When the sequence length was greater than 25, the error increased, given the limited data; thus, the deep learning model was insufficiently trained. The coverage of the data was also reduced as the sequence length increased. The MAE value of the model with one cardiac cycle was 4.05 and 3.31 mmHg for SBP and DBP, respectively, which is larger than the model with 10 cardiac cycles. However, the coverage at the sequence length of one was 81%, whereas the model with 10 cardiac cycles covered 59% of the data.

Finally, the limitation of this study is briefly discussed. The data of patients with hypertension were not included in the study. However, about 8% and 2% of the BP data were in hypertension stage 1 and stage 2 ranges, though no subject was diagnosed as a hypertension patient. According to the guidelines for BP classification in adults, BP can be classified as: Normal, Prehypertension, Stage 1 Hypertension, and Stage 2 Hypertension [24]. The evaluation results of classification performance for hypertension are shown in Table 10. The total accuracy for hypertension classification was 81% and 89% for SBP and DBP respectively.

## 5. Conclusions and Future Work

In this paper, we have proposed a beat-to-beat continuous BP estimation algorithm with a feature-based LSTM network using the features from ECG, PPG, and BCG. The result showed that the performance was improved with the BCG signal, and the feature-based network outperformed the raw signal-based network. In addition, a generalized model was considered with LOSO analysis, and a multi-day test was performed to evaluate the model reproducibility. Moreover, the results demonstrated that the LOSO model with fine-tuning was better than the personalized model in the multi-day test. In future studies, the data of comprising subjects diagnosed with hypertension will be incorporated to produce a more generalized model. In addition, the BCG signal used in this study can be measured unobtrusively on everyday surfaces like chairs and beds; in our future work, we intend to pursue the development of unobtrusive BP estimation methods.

## Figures and Tables

**Table 1:** List of features used as inputs in our model.

Feature	Description
R-R Interval (RRI)	ECG R-peak to R-peak interval
Pulse Transit Time (PTT)	ECG R-peak to dPPG peak interval
R-J Interval (RJI)	ECG R-peak to BCG J-peak interval
I-P Interval (IPI)	BCG I notch to dPPG peak interval
ECGamp	Amplitude of ECG R-peak
BCGamp	Amplitude of BCG J-peak
PPGamp	Amplitude of dPPG peak

**Table 2:** Feature list of different inputs.

Inputs	Features
ECG	RRI, ECGamp
ECG, BCG	RRI, RJI, ECGamp, BCGamp
ECG, PPG	RRI, PTT, ECGamp, PPGamp
ECG, PPG, BCG	RRI, PTT, RJI, IPI, ECGamp, BCGamp, PPGamp

**Table 3:** Mean values of mean absolute error (MAE), root mean squared error (RMSE), and correlation coefficient (CC) for different inputs of personal-specific model.

Inputs	SBP			DBP		
	MAE	RMSE	CC	MAE	RMSE	CC
ECG	3.81	4.75	0.50	2.70	3.40	0.51
ECG, BCG	3.50	4.42	0.59	2.51	3.15	0.62
ECG, PPG	2.84	3.57	0.74	2.29	2.88	0.70
**ECG, PPG, BCG**	**2.62**	**3.36**	**0.77**	**2.03**	**2.57**	**0.76**

**Table 4:** Mean values of MAE, RMSE, and CC for each model.

Model	Systolic Blood Pressure(SBP)			Diastolic Blood Pressure (DBP)		
	MAE	RMSE	CC	MAE	RMSE	CC
Personal	2.62	3.36	0.77	2.03	2.57	0.76
LOSO	10.01	11.26	0.40	5.64	6.52	0.40
Tuned LOSO	2.56	3.25	0.80	2.05	2.61	0.76

**Table 5:** Mean MAE, RMSE, and CC values of inter-visit test analysis.

Train	Test	Model	SBP			DBP		
			MAE	RMSE	CC	MAE	RMSE	CC
Visit #1	Visit #2	Personal	7.12	8.99	0.41	6.22	7.61	0.38
		LOSO	10.23	11.49	0.41	5.94	6.81	0.41
		Tuned LOSO	**5.81**	**6.78**	**0.53**	**5.34**	**6.14**	**0.51**
Visit #2	Visit #1	Personal	6.23	7.60	0.45	5.20	6.27	0.41
		LOSO	10.82	11.97	0.45	6.17	7.06	0.41
		Tuned LOSO	**5.84**	**6.85**	**0.52**	**5.14**	**5.97**	**0.49**
Total		Personal	6.67	8.29	0.43	5.71	6.94	0.40
		LOSO	10.52	11.73	0.43	6.06	6.94	0.41
		Tuned LOSO	**5.82**	**6.82**	**0.53**	**5.24**	**6.06**	**0.50**

**Table 6:** Performance evaluation using British Hypertension Society (BHS) standard.

		Cumulative Absolute Error Percentage			Grade
		≤5 mmHg	≤10 mmHg	≤15 mmHg	
BHS standard		60%	85%	95%	A
		50%	75%	90%	B
		40%	65%	85%	C
		Worse than C			D
Proposed model (one-day test)	SBP	89.3%	99.4%	100.0%	A
	DBP	94.7%	99.8%	100.0%	A
Proposed model (multi-day test)	SBP	51.6%	81.4%	96.3%	B
	DBP	56.1%	87.9%	98.3%	B

**Table 7:** Performance evaluation using Advancement of Medical Instrumentation (AAMI) standard.

		ME (mmHg)	STD (mmHg)
AAMI Standard		≤5	≤8
Proposed model (one-day test)	SBP	−0.09	3.15
	DBP	−0.07	2.52
Proposed model (multi-day test)	SBP	−0.07	7.30
	DBP	−0.17	6.4

**Table 8:** Comparison with other methods.

Model	Equation	MAE (mmHg)	
		SBP	DBP
Chen et al. [5]	$SBP=SBP_{0}-\frac{2}{\gamma PTT_{0}}\left(PTT-PTT_{0}\right)$	4.32	-
Poon et al. [6]	$SBP=MBP_{0}+\frac{2}{\gamma }\ln\left(\frac{PTT_{0}}{PTT}\right)+\frac{2}{3}\cdot PP_{0}\cdot {\left(\frac{PTT_{0}}{PTT}\right)}^{2}$ $DBP=MBP_{0}+\frac{2}{\gamma }\ln\left(\frac{PTT_{0}}{PTT}\right)\cdot \frac{1}{3}\cdot PP_{0}\cdot {\left(\frac{PTT_{0}}{PTT}\right)}^{2}$	4.70	3.28
Ding et al. [7]	$SBP=DBP_{0}\cdot \frac{PIR_{0}}{PIR}+PP_{0}\cdot {\left(\frac{PTT_{0}}{PTT}\right)}^{2}$ $DBP=DBP_{0}\cdot \frac{PIR_{0}}{PIR}$	4.47	3.15
MLR model (w/o BCG features)	$SBP=a_{1}+b_{1}\cdot PTT+c_{1}\cdot RRI$ $DBP=a_{2}+b_{2}\cdot PTT+c_{2}\cdot RRI$	4.25	3.16
MLR model (with BCG features)	$SBP=a_{1}+b_{1}\cdot PTT+c_{1}\cdot RRI+d_{1}\cdot RJI+e_{1}\cdot IPI$ $DBP=a_{2}+b_{2}\cdot PTT+c_{2}\cdot RRI+d_{2}\cdot RJI+e_{2}\cdot IPI$	4.17	3.12
MLR model (with previous features)	$SBP=a_{1}+\sum_{N}(b_{1i}\cdot PTT_{i}+c_{1i}\cdot RRI_{i}+d_{1i}\cdot RJI_{i}+e_{1i}\cdot IPI_{i})$ $DBP=a_{2}+\sum_{N}(b_{2i}\cdot PTT_{i}+c_{2i}\cdot RRI_{i}+d_{2i}\cdot RJI_{i}+e_{2i}\cdot IPI_{i}$)	3.71	2.65
**Proposed model**	Long short-term memory (**LSTM)**	**2.62**	**2.03**

**Table 9:** Comparisons with related works.

Author	Dataset	Model	Input	Validation Method	SBP Error (mmHg)		DBP Error (mmHg)	
					MAE	RMSE	MAE	RMSE
Kachuee et al. [19]	N = 1000 10 min (MIMIC III)	AdaBoost	ECG, PPG features	Personal	8.21		4.31	
				10-fold cross validation	11.17		5.35	
Slapničar et al. [20]	510 subjects 700 h (MIMIC III)	ResNet	Raw PPG	LOSO	15.41		12.38	
				Tuned LOSO	9.43		6.88	
Hsu et al. [21]	N = 9000 (MIMIC II)	DNN	PPG features	10-fold cross-validation	3.21	4.63	2.23	3.21
Wu et al. [14]	N = 85	DNN	ECG, PPG features	10-fold cross-validation	3.31	4.60	2.22	3.15
Su et al. [23]	N = 84 10 min	Bi-LSTM	ECG, PPG features	Personal (one-day)		3.73		2.43
				Personal (multi-day)		5.81		5.21
Previous work [16]	N = 15 30 min	CNN, Bi-GRU, attention	Raw ECG, PPG, BCG	Personal	4.06	5.42	3.33	4.30
**Proposed work**	**N = 18** **30 min**	**Bi-LSTM**	**ECG, PPG, BCG features**	**Tuned LOSO** **(one-day)**	**2.56**	**3.25**	**2.05**	**2.61**
				**Tuned LOSO** **(multi-day)**	**5.82**	**6.82**	**5.24**	**6.06**
				**LOSO**	**10.01**	**11.26**	**5.60**	**6.52**

**Table 10:** Hypertension classification accuracies for SBP and DBP.

BP Class	SBP		DBP	
	Range (mmHg)	Accuracy	Range (mmHg)	Accuracy
Normal	BP < 120	84%	BP < 80	90%
Prehypertension	120≤ BP < 130	82%		
Stage 1 Hypertension	130≤ BP < 140	97%	80≤ BP < 90	89%
Stage 2 Hypertension	BP≥140	98%	BP≥90	100%
Total		81%		89%

## Data Availability

Data sharing is not applicable to this article.

## References

[1] Ogedegbe G., Pickering T. (2010). Principles and techniques of blood pressure measurement. Cardiol. Clin..

[2] Yoo S., Baek H., Doh K., Jeong J., Ahn S., Oh I.Y., Kim K. (2018). Validation of the mobile wireless digital automatic blood pressure monitor using the cuff pressure oscillometric method, for clinical use and self-management, according to international protocols. Biomed. Eng. Lett..

[3] Ding X., Zhang Y.T. (2019). Pulse transit time technique for cuffless unobtrusive blood pressure measurement: From theory to algorithm. Biomed. Eng. Lett..

[4] Wong M.Y., Poon C.C., Zhang Y.T. (2009). An evaluation of the cuffless blood pressure estimation based on pulse transit time technique: A half year study on normotensive subjects. Cardiovasc. Eng..

[5] Chen W., Kobayashi T., Ichikawa S., Takeuchi Y., Togawa T. (2000). Continuous estimation of systolic blood pressure using the pulse arrival time and intermittent calibration. Med. Biol. Eng. Comput..

[6] Poon C., Zhang Y. Cuff-less and noninvasive measurements of arterial blood pressure by pulse transit time. Proceedings of the 2005 IEEE Engineering in Medicine and Biology 27th Annual Conference.

[7] Ding X.R., Zhang Y.T., Liu J., Dai W.X., Tsang H.K. (2016). Continuous Cuffless Blood Pressure Estimation Using Pulse Transit Time and Photoplethysmogram Intensity Ratio. IEEE Trans. Biomed. Eng..

[8] Payne R.A., Symeonides C.N., Webb D.J., Maxwell S.R. (2006). Pulse transit time measured from the ECG: An unreliable marker of beat-to-beat blood pressure. J. Appl. Physiol..

[9] Shin J.H., Lee K.M., Park K.S. (2009). Non-constrained monitoring of systolic blood pressure on a weighing scale. Physiol. Meas..

[10] Lee K.J., Roh J., Cho D., Hyeong J., Kim S. (2019). A Chair-Based Unconstrained/Nonintrusive Cuffless Blood Pressure Monitoring System Using a Two-Channel Ballistocardiogram. Sensors.

[11] Yildirim O., Plawiak P., Tan R.S., Acharya U.R. (2018). Arrhythmia detection using deep convolutional neural network with long duration ECG signals. Comput. Biol. Med..

[12] Dey D., Chaudhuri S., Munshi S. (2018). Obstructive sleep apnoea detection using convolutional neural network based deep learning framework. Biomed. Eng. Lett..

[13] Rundo F., Conoci S., Ortis A., Battiato S. (2018). An Advanced Bio-Inspired PhotoPlethysmoGraphy (PPG) and ECG Pattern Recognition System for Medical Assessment. Sensors.

[14] Wu D., Xu L., Zhang R., Zhang H., Ren L., Zhang Y.-T. (2018). Continuous cuff-less blood pressure estimation based on combined information using deep learning approach. J. Med. Imaging Health Inform..

[15] Li Y.H., Harfiya L.N., Purwandari K., Lin Y.D. (2020). Real-Time Cuffless Continuous Blood Pressure Estimation Using Deep Learning Model. Sensors.

[16] Eom H., Lee D., Han S., Hariyani Y.S., Lim Y., Sohn I., Park K., Park C. (2020). End-to-End Deep Learning Architecture for Continuous Blood Pressure Estimation Using Attention Mechanism. Sensors.

[17] Hochreiter S., Schmidhuber J. (1997). Long short-term memory. Neural Comput..

[18] O’Brien E., Petrie J., Littler W., de Swiet M., Padfield P.L., O’Malley K., Jamieson M., Altman D., Bland M., Atkins N. (1990). The British Hypertension Society protocol for the evaluation of automated and semi-automated blood pressure measuring devices with special reference to ambulatory systems. J. Hypertens.

[19] Kachuee M., Kiani M.M., Mohammadzade H., Shabany M. (2017). Cuffless Blood Pressure Estimation Algorithms for Continuous Health-Care Monitoring. IEEE Trans. Biomed. Eng..

[20] Slapničar G., Mlakar N., Lustrek M. (2019). Blood Pressure Estimation from Photoplethysmogram Using a Spectro-Temporal Deep Neural Network. Sensors.

[21] Hsu Y.C., Li Y.H., Chang C.C., Harfiya L.N. (2020). Generalized Deep Neural Network Model for Cuffless Blood Pressure Estimation with Photoplethysmogram Signal Only. Sensors.

[22] Johnson A.E.W., Pollard T.J., Shen L., Lehman L.-W.H., Feng M., Ghassemi M., Moody B., Szolovits P., Anthony Celi L., Mark R.G. (2016). MIMIC-III, A freely accessible critical care database. Sci. Data.

[23] Su P., Ding X.-R., Zhang Y.-T., Liu J., Miao F., Zhao N. Long-term blood pressure prediction with deep recurrent neural networks. Proceedings of the IEEE EMBS International Conference on Biomedical & Health Informatics (BHI).

[24] Whelton P.K., Carey R.M. (2018). The 2017 American College of Cardiology/American Heart Association Clinical Practice Guideline for High Blood Pressure in Adults. JAMA Cardiol..

